# Clinical Trials Radiographers identifying priority challenges associated with implementing a national programme of clinical trials in the United Kingdom’s first proton beam therapy centre

**DOI:** 10.1093/bjro/tzae012

**Published:** 2024-05-23

**Authors:** Lucy S C Davies, Louise McHugh, Sally Falk, Jacqui Bridge, Philip F Amaro, Lee Whiteside, Rachael Bailey, Julie Webb, Cynthia L Eccles

**Affiliations:** Department of Radiotherapy, The Christie NHS Foundation Trust, Manchester M20 4BX, United Kingdom; Department of Radiotherapy, The Christie NHS Foundation Trust, Manchester M20 4BX, United Kingdom; Proton Beam Therapy, The Christie NHS Foundation Trust, Manchester M20 4BX, United Kingdom; Department of Radiotherapy, The Christie NHS Foundation Trust, Manchester M20 4BX, United Kingdom; Department of Radiotherapy, The Christie NHS Foundation Trust, Manchester M20 4BX, United Kingdom; Department of Radiotherapy, The Christie NHS Foundation Trust, Manchester M20 4BX, United Kingdom; Department of Radiotherapy, The Christie NHS Foundation Trust, Manchester M20 4BX, United Kingdom; Department of Radiotherapy, The Christie NHS Foundation Trust, Manchester M20 4BX, United Kingdom; Department of Radiotherapy, The Christie NHS Foundation Trust, Manchester M20 4BX, United Kingdom; Division of Cancer Sciences, School of Medical Sciences, Faculty of Biology, Medicine and Health, The University of Manchester, Manchester Academic Health Science Centre, Manchester M13 9NT, United Kingdom

**Keywords:** proton beam therapy, clinical trials, nominal group technique

## Abstract

**Objectives:**

This article is an evaluation of the current trial processes within a national proton beam therapy (PBT) clinical trial service in the United Kingdom. The work within the article identifies priority challenges associated with the implementation of PBT trials with a view to improving patient trial processes.

**Methods:**

The nominal group technique (NGT) was used. Five Clinical Trials Radiographers were asked the target question “what are the major challenges when implementing PBT clinical trials and facilitating PBT trial-related activities?” Participants individually and silently listed their challenges to the target question. Following this, group discussion clarified and refined responses. Participants then individually selected five challenges that they deemed most pertinent to the target question, giving a weighted score (out of 10). Individual scores were combined to provide a ranked, weighted order of challenges. Further group discussion identified improvement strategies to the highest scored challenges.

**Results:**

After combining lists generated by participants, 59 challenges were identified. Group discussion eliminated 27 responses. Eighteen were merged, resulting in 14 challenges. The two challenges that ranked highest were: (i) lack of initial understanding of the responsibilities of teams and who the relevant stakeholders were, and (ii) that a national PBT service requires the provision of shared care across multi-disciplinary teams and sites. Improvement areas include the development of shared protocols, clarifying stakeholder responsibilities and improving communication between centres to streamline PBT trial processes.

**Conclusions:**

This work has identified priority areas requiring development to improve the conduct of a national PBT clinical trials programme.

**Advances in knowledge:**

This is the first publication to evaluate current clinical trial processes for the United Kingdom’s PBT service.

## Introduction

The United Kingdom’s first of only two National Health Service (NHS) high-energy proton beam therapy (PBT) centres (at The Christie NHS Foundation Trust, Manchester) became clinically operational in 2018. The second centre (at University College London NHS Foundation Trust, London) treated their first patient in 2021. The national PBT service in the United Kingdom has an agreed national agenda of specific indications, determined by NHS England, reflecting diagnoses considered to have a reasonable evidence base.^1^ A key objective of the United Kingdom’s PBT service is to contribute to the research and evidence to support the clinical use of PBT.^2^ Other indications are offered under clinical trials—national, international, or investigator-led to evaluate their efficacy. To this end, a significant proportion of patients referred nationally for PBT have been enrolled in clinical trials. The multi-faceted workflow in a newly established national PBT service can present challenges. Unlike existing photon radiotherapy trial pathways, which have established local referral routes, there is added complexity in PBT trials because of the emphasis on external patient referrals from health care organisations across the whole of the United Kingdom. Consequently, this warrants a need for greater research governance requirements and the streamlining of complex PBT data transfer procedures.

Referrals for PBT to one of the two PBT centres are initiated by the Consultant Clinical Oncologist following discussion at the relevant multi-disciplinary team (MDT) meeting, at the patient’s local NHS cancer centre (ie, the patient’s “home” centre) using the NHS PBT Referral Portal.^3^ The referral is reviewed by a panel of experts (made up of leading Radiologists, Surgeons, and Clinical and Medical Oncologists from across the United Kingdom) prior to acceptance for PBT.^2^ For patients participating in, or considering entry into a clinical trial, the “home” centre maintain overarching responsibility for the consideration of entry, consent, and randomisation (when required) of patients in the trial. In certain circumstances (eg, some paediatric cases), the “home” centre that initially referred the patient for PBT may not be open to recruitment for the clinical trial. In these instances, the patient may be referred to another NHS cancer centre that is actively recruiting to allow enrolment into the appropriate trial. This can mean several organisations are involved in patient trial activity, resulting in complex patient pathways requiring the provision of shared care across multiple MDTs and health care systems.

As UK-based proton therapy clinical trials have come online as part of a new national service, there is a need to systematically evaluate and consider issues and challenges to patient trial pathways. The work presented in this paper prioritised challenges of introducing a national PBT clinical trials programme at the United Kingdom’s first PBT centre, according to experienced Clinical Trials Radiographers using an evaluative methodology—the nominal group technique (NGT). NGT is a systematic, brainstorming tool for quality improvement.^4^ Similar to a Delphi survey,^5^ the NGT is an example of a formal consensus building approach involving participants who are expert, experienced, or knowledgeable, in the issue under investigation.^6^ A key feature of the NGT approach is the use of a highly structured small group discussion to elicit and prioritise responses to a specific question.

Whilst it is important to acknowledge the conduct of PBT clinical trials are carried out as part of an MDT with expertise input from many disciplines, the focus of this evaluation is centred on the Clinical Trials Radiographer role. In this instance, Clinical Trials Radiographers are experienced in PBT delivery and are responsible for liaising with the relevant teams from the “home” centres to manage research governance and co-ordinate trial activity throughout the entire PBT patient pathway. Research activity includes patient-facing tasks such as adverse event monitoring throughout treatment delivery, the reporting of PBT trial data collection in accordance with trial-specific Radiotherapy Quality Assurance guidance, and subsequently, the secure transfer of all necessary trial data back to the referring “home” centre or relevant Clinical Trials Unit (CTU). The Clinical Trials Radiographer’s role therefore provides a unique insight into the challenges presented by the pathway.

The first aim of this work was to use the NGT to systematically identify key challenges and issues surrounding the practical implementation of PBT clinical trials. The second was to make recommendations informing local operational policy for the conduct of PBT clinical trials.

## Methods

This work was undertaken as a service evaluation, approved by the local quality improvement and clinical audit committee. The NGT workshop was carried out in September 2021 as a singular session of approximately 3.5 h. A panel was convened comprising five Clinical Trials Radiographers. The Clinical Trials Radiographers had varying acquired experience in both radiotherapy modalities (photon 1-48 months and proton 1-24 months), facilitating the implementation of PBT clinical trials, patient pathways, and the reporting of treatment data in adherence with approved trial protocols. The panel was facilitated by the first author.

The NGT, adapted from Potter et al,^4^ was used to identify priority challenges, barriers, and perspectives of facilitating a national PBT clinical trial programme. The NGT process involved presentation of a target question to the panel, followed by *silent generation of ideas*, *round robin*, *group discussion*, *scoring,* and *data combining* phases. Consensus was reached on which challenges require prioritisation. Table 1 outlines the NGT protocol followed in this work.

**Table 1.** The nominal group technique (NGT) protocol used in this project.

The nominal group technique protocol		
		Time allocated (min)
**Step 1**	**Introduction** Following a short introduction to the NGT, participants were presented with the target question: “What are the major challenges when implementing PBT clinical trials and facilitating PBT trial-related activities?”	10-15
**Step 2**	**Silent phase** Participants in the group were asked to silently, without conferring, record their responses to the subject question on a blank Microsoft Word document. There was no limit to the number of items that could be listed.	15-30
**Step 3**	**Round robin phase** Ideas that were generated individually were fed back to the larger group, where the panel facilitator recorded each of the individual responses from all participants onto a Microsoft Excel spreadsheet.	30-45
**Step 4**	**Discussion/clarification phase** All items at this stage were projected onto a large screen. Through group discussion, the list of responses was clarified. Where appropriate, related items were merged, and duplications were eliminated. This process generated a refined list.	30-45
**Step 5**	**Scoring and ranking phase** Each participant was provided with a copy of the refined list, from which participants were asked to individually prioritise five responses by giving a weighted score. A total of 10 points were available for each participant to distribute to their five priority responses.	15-30
**Step 6**	**Large group data combining phase** The group reconvened, and the individual scores were combined to the ranked, weighted responses of the cohort to the target question.	15-30

Source: Adapted from Potter et al.^4^

Abbreviations: NGT = nominal group technique, PBT = proton beam therapy.

The panel facilitator started the NGT process by giving a brief introduction of the purpose and procedure of the meeting. The facilitator then posed the target question “what are the major challenges when implementing PBT clinical trials and facilitating PBT trial-related activities?” Participants had access to individual laptops and were asked to silently, without conferring, write their challenges to the implementation and facilitation of PBT trials on a blank Microsoft Word 2010 (Microsoft, Redmond, WA, United States) document. No limit to the number of items a participant could list was given. Ideas that were generated individually were fed back to the larger group in a *round robin* dialogue. Each participant emailed their responses to the panel facilitator who transcribed each of the items onto Microsoft Excel 2010 (Microsoft, Redmond, WA, United States). This was projected onto a large screen visible to all participants. The group then discussed the list of challenges providing clarification, merged related items (where appropriate), and removed any duplicate challenges. This generated an agreed, refined list of challenges. From this refined list, participants were asked to individually select the top five challenges that they deemed most pertinent to the target question. Participants gave a weighted score (out of 10 points) to distribute amongst their five chosen challenges. Finally, the individual scores were combined for the larger group providing an overall ranked, weighted order of challenges to the target question.

The ranked challenges were characterised as “priorities”.^7^ Following generation of the list of “priorities”, further discussion took place to identify potential solutions and improvement strategies to the highest scored priorities.

## Results

In response to the target question put to the panel “what are the major challenges when implementing PBT clinical trials and facilitating PBT trial-related activities?”, the *silent* individual and *round robin* group brainstorming phases yielded a total of 59 responses.

**Figure 1.**
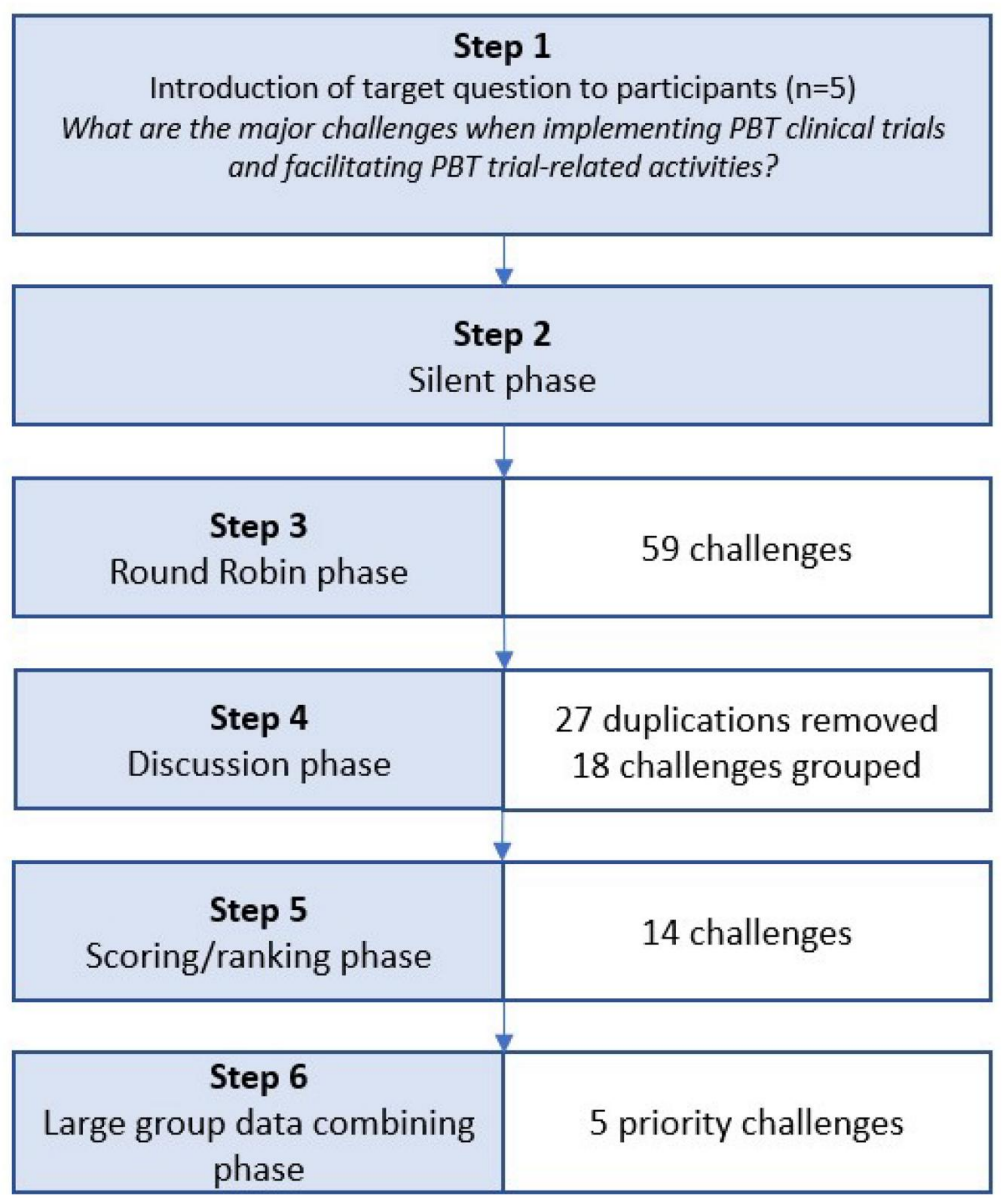
Flowchart of results to the NGT protocol.

The panel, following group discussion, clarified and refined the 59 responses. Eighteen related items were grouped together. Twenty-seven duplications were eliminated, resulting in 14 responses (Figure 1). Of these 14 responses, participants were tasked with individually selecting five responses that they deemed most pertinent to the target question. A score (out of 10 points) was distributed across their five selected challenges, producing a weighted order of responses.

In the final step of the NGT process, the individual weighted responses were combined. Where participants had selected the same responses, the points allocated to each data item was totalled. Combining of the lists generated a ranked, weighted order of responses from the cohort to the target question (Table 2).

**Table 2.** Weighted rank order of challenges.

Responses to target question	Participants					Total points	Priority challenge
	1	2	3	4	5		
Lack of awareness of the key stakeholders and MDTs (internal and external to the PBT centre) involved, including the understanding of the respective responsibilities	4	3		3	2	**12**	1
National PBT service: increased complexity of the flow of trial data and communication across the multiple recruiting and referring sites, trial centre, and local teams	1		3	3	4	**11**	2
Other stakeholders’ awareness of the Research Radiographer team and the team’s visibility and presence	2	2	1		2	**7**	3
Funding for trials staffing and under resourcing of PBT trial dedicated individuals	1	1	1	1	1	**5**	4
Trying to fit the PBT trial pathway into existing photon trial pathways where the model and trial-related tasks may differ			3	2		**5**	5
Unfamiliarity of the PBT patient pathway including the referral process of the patient prior to attending the PBT centre	2				1	**3**	
Internal PBT processes and another record and verify system to consider		2		1		**3**	
Patient pathway when care has begun at the PBT centre (site assessment visit week including clinic appointments, pre-treatment sessions, other trial-related appointments)		2				**2**	
Ethical considerations (data sharing, anonymisation and pseudonymised data, ensuring documented informed consent)			2			**2**	
Preparing for contingency when PBT machine breakdown occurs (treating with photons and the trial implications of this)						**0**	
New local documentation/policies/work instructions that need to be written for the quality system						**0**	
Dissemination of the relevant clinical trial information to multiple teams within PBT						**0**	
PBT trial data upload responsibilities—initially unaware of how much data would be required to be sent and who was responsible for exporting/anonymising of radiotherapy-specific data (planning scans, structure set, plan, on-treatment cone-beam CT data)						**0**	
Trial Case Report Form completion—forms for data collection are not proton specific (eg, reporting of PBT unit of dose/proton energy), including contacting each trial sponsor to feedback.						**0**	

Abbreviations: MDT = multi-disciplinary team, PBT = proton beam therapy.

Five priority challenges were generated (Table 2). Of these, two challenges were weighted notably higher than the others. These were:

1. the lack of awareness of the key stakeholders and MDTs (internal and external to the PBT centre) involved in the PBT trial process, including the understanding of the respective responsibilities.
2. that the national PBT service requires shared care across multiple sites and MDTs, increasing the complexity of the flow of trial data and communication between organisations.

The results of the NGT, that is, the ranked “priorities” were used to identify areas for development, and improvement to inform potential changes to local operational policies and processes. Based on these priorities, the group proposed four key areas to focus on to reduce the challenges presented by this multi-site, multi-disciplinary national trials programme. Table 3 outlines the proposed areas for improvement generated from the discussion by the panel.

**Table 3.** Improvement areas.

Improvement areas
Development of shared protocols and working processes, clarifying responsibilities of local teams Establishment of disease-specific PBT trial groups involving the MDT Creation of shared learning materials to all PBT staff groups describing the key points of each new PBT trial and implications to practice
Improve the communication between investigator, trial recruiter, and trial—delivery centre Build a core directory of key PBT trial contacts for each “home” and recruiting centre to direct trial queries to
Streamline PBT data transfer process When contact has been established with “home” or recruiting centre for trial information, request relevant trial documentation as early as possible in the patient trial pathway Keep in continual contact outlining key dates when trial data reported at the PBT centre will be transferred back to the trial recruiter or CTU
Establish communication networks with Radiotherapy Quality Assurance teams Include patient timelines and anticipated dates for quality assurance reviews in communication when trial-mandated prospective review is required before commencing PBT treatment

Abbreviations: CTU = Clinical Trials Unit, MDT = multi-disciplinary team, PBT = proton beam therapy.

## Discussion

This manuscript describes the first evaluation of implementing a national programme of clinical trials by Clinical Trials Radiographers within a PBT service, with a view to improving patient and trials pathways across the United Kingdom. The overall landscape of PBT national trials is not entirely coterminous, in part because of the need for multiple stakeholders and different organisations to be involved. Additionally, it is a relatively new and rapidly developing service. The justification for undertaking this work was to attempt to introduce more cohesion into the PBT clinical trials process, by identifying the key *priority* challenges. The rationale for the systematic application of the NGT process was to distil down the initial wide ranging brainstorming issues (ie, in this case, 59) to identify the issues with highest priority as determined as a result of expert opinion and dialogue from a group of Clinical Trials Radiographers. In this case, five priority areas were identified, and this paper outlines the process in which these were arrived at.

All five priority challenges are to some extent interrelated. In particular, the two highest ranking challenges (lack of awareness of the key stakeholders and MDTs, and the increased complexity of the flow of trial data and communication across the multiple recruiting and referring sites, trial centre, and local teams), which were prioritised as development areas, are closely associated. “Priority” one refers to the *understanding* of individuals’ and teams’ responsibilities, and “priority” two, the *practicality* of working across multiple organisations.

The first challenge identified was the initial lack of awareness of all the key stakeholders and teams (both internal and external to the PBT centre) involved in the PBT trial process, including understanding the respective responsibilities. Intrinsic to the implementation of the national PBT service was the establishment of complex patient pathways, development of workflows and processes outside of known local channels, and the upskilling and training of the MDTs involved in administering care and treatment. At the time of conducting this work, the first PBT centre to open was in the early years of clinical operation, with the second PBT centre not yet in clinical operation. The MDTs and working processes in PBT were still in their relative infancy and undergoing expansion and continual development. The Clinical Trials Radiographer team, despite being experienced in the conduct of photon radiotherapy trials and having knowledge of established local referral routes and patient pathways, had to undergo a steep learning process to understand the set-up and framework within the new national PBT service, and the teams that would be involved in carrying out trial-specific activity. Whilst the Chief Investigator retains overall responsibility for the conduct of all trial activities,^8^ the delegation of trial duties and the clarification of respective responsibilities to the appropriate research teams (internal and external of the PBT centre) still need to be agreed. Internally, this can involve establishing which local research team within the PBT centre are responsible for the reporting of individual trial-specified tasks (for instance, pre-treatment and on-treatment activities such as Radiotherapy Quality Assurance, and end of treatment assessments). Also pertinent is the clarification of the responsibilities of key external individuals and organisations. Considerations of the responsibilities of both the PBT and “home” teams’ links to the second ranked challenge of communication of trial information to all relevant stakeholders.

In relation to communication between stakeholders, there is an additional complexity when patients are referred for PBT while enrolled in or considering entry into a clinical trial. Trial conduct must comply with the approved protocol and adhere to International Conference on Harmonization-Good Clinical Practice and Research Governance guidelines.^9^ The Clinical Trials Radiographer team at the PBT centre are responsible for obtaining the essential trial documentation (eg, study consent form and/or randomisation results) from either the recruiting or “home” centres and communicating the information to local teams to ensure PBT is planned and delivered as mandated by trial protocol. Similarly, when the patient has finished PBT, trial-mandated data (for instance, trial Case Report Forms [CRFs]) detailing radiotherapy treatment or adverse event CRFs must be securely transferred back to the recruiting centre. Because the PBT centre provides a national service, the provision of care is shared across numerous MDTs and multiple centres, often, with each organisation having their own working processes. This multistakeholder approach increases the complexity of the flow of trial data and communication across the multiple teams involved in the care of the patient. Undoubtedly, establishing effective communication links and subsequent routes of data transfer with key research contacts at the recruiting sites will aid in streamlining this process. A comparable theme emphasising the importance of building constructive relationships and strong pathways of communication between research teams and recruiting sites has previously been reported. This was evidenced in a collaborative study by the National Cancer Research Institute Clinical and Translational Radiotherapy Research Working Group and the National Institute for Health Research where the barriers and potential solutions in the set-up of photon radiotherapy trials in the United Kingdom were identified.^10^

The second aim of this work was to make recommendations based on the results of answering the target question. These recommendations were derived from the results of the NGT. A number of development areas were proposed in response to this evaluative project. Table 3 outlines the improvement areas to address the challenges identified by the panel. Since this work was originally carried out, all improvement areas have been actioned and commenced implementation. This process of systematically identifying and addressing the priority challenges of the practical implementation of PBT trials should support further refinement of the conduct of PBT trials in the United Kingdom and drive forwards the United Kingdom’s national PBT service as a key contribution to the evaluation of the efficacy PBT through clinical studies.^11^

Using the evaluative NGT methodology in this work had several benefits. Consensus building methods can be particularly useful in areas where the evidence base is poor or non-existent, and where unanimity of professional opinion has not yet been established.^12^ NGT was first described in the 1960s as an organised planning technique to facilitate effective group decision-making in social psychological research.^13^ Since then, it has subsequently been used as a technique for developing clinical guidelines,^14^ as an approach to evaluate user involvement in health service delivery,^12^ and as an appropriate methodology for health services research.^10^ Structuring the process provides a safe means for participants to contribute but equally allows for more creative responses, and the contribution of ideas that may not be expressed in other research processes. The NGT provides a tool for facilitating individuals who are ordinarily less vocal in group discussions. The discussion and clarification phase helps to provide the wider context from which responses that have been identified individually have emerged. The wider group participation helps clarify suggestions from other group members’ experiences. Data analysis of the NGT encompasses both qualitative and quantitative methods, therefore providing a systematic process. Furthermore, the collaborative nature of the NGT increases participants’ ownership of the research and therefore increases the likelihood of changing clinical practice and policy.^15^

There were several limitations of this study. The participant sample centred on the experiences of a singular professional role (ie, the Clinical Trials Radiographer) determining preliminary findings before presenting within a national MDT for further and deeper investigation. Thus, it is acknowledged that this work does not consider the evaluation of the whole MDT involved in the patient care, nor the entirety of the trial patient pathway. The inclusion of other stakeholders involved in PBT trial implementation and the patient pathway (eg, Oncologists, Research Nurses, Medical Physicist) could have influenced the data generated and was not considered necessary within the scope of this paper, as the aim was to target it from the unique Clinical Trials Radiographer team role. A small number of participants took part in the NGT. However, this afforded the panel facilitator with a manageable group whilst also allowing for the generation of a range of opinions and ideas. There is much discussion as to what constitutes the optimum number of participants, with Delbecq and Van de Ven^7^ recommending NGT groups should be ideally made up of no more than 5-9 participants; however, larger numbers of more than 200 can be accommodated. Nevertheless, the number of Clinical Trials Radiographers with acquired experience in the facilitation and conduct of national PBT clinical trials was an insurmountable limiting factor in sample size.

## Conclusions

The implementation of PBT clinical trials and facilitation of the patient pathway can be complex. This work has identified key challenges experienced by a research team tasked with carrying out the research governance aspects of PBT trial conduct during the initial stages of facilitating a national PBT clinical trial programme. In doing so, it has highlighted the need to develop shared protocols identifying and clarifying responsibilities of multiple stakeholders to streamline PBT trial processes, promote understanding of the respective responsibilities, and improve communication and collaborative working between centres referring patients for PBT and PBT trials. NGT is an effective tool for reaching consensus and identifying context-specific priority areas for quality improvement. Further multistakeholder, collaborative work is ongoing, continually reviewing the PBT trial processes across both PBT centres to support the continued delivery of a national PBT clinical trials programme.

## Funding

Cynthia L. Eccles is supported by NIHR Manchester Biomedical Research Centre (NIHR203308).

## Conflicts of interest

None declared.
